# Social Media Use and Sleep Quality in Adolescents and Young Adults: A Scoping Review of Reviews

**DOI:** 10.3390/children13010051

**Published:** 2025-12-30

**Authors:** Awele Ndubisi, Felix Agyapong-Opoku, Belinda Agyapong

**Affiliations:** 1 School of Medicine, University of Galway, H91 TK33 Galway, Ireland; 2 Department of Psychiatry, University of Alberta, Edmonton, AB T6G 2H5, Canada

**Keywords:** social media, adolescents, sleep quality, scoping review

## Abstract

**Background:** Social media use has grown rapidly and has been integrated into the lives of many adolescents and young adults worldwide. Research indicates that excessive social media engagement can negatively impact sleep quality through various mechanisms. **Objective:** This scoping review of reviews aims to explore the relationship between social media use and sleep quality among adolescents and young adults, synthesize existing evidence, identify research gaps, and highlight directions for future research. **Methods:** Arksey’s and O’Malley’s five-stage framework was used to conduct this scoping review. Searches were conducted in PubMed, Web of Science, Embase, Medline, and Scopus for articles published between 2020 and 2025. The inclusion criteria were systematic reviews or meta-analyses focused on adolescents and young adults, examining social media use in relation to sleep quality, and peer-reviewed articles written in English. Ten articles met all eligibility criteria and were included in the review. **Results:** The findings indicate a small but consistent negative effect of social media use on sleep quality. Problematic social media use showed a stronger association with poorer sleep than general social media use. Specific platforms such as Facebook and Twitter contributed most to shorter sleep duration, later bedtimes, and poorer sleep quality, while Snapchat and Instagram showed moderate effects, and WhatsApp and WeChat showed smaller effects. **Conclusions:** Problematic social media use is strongly associated with poorer sleep quality, while general use may have smaller effects. Future research focusing on longitudinal studies would help deepen the understanding of the effects of social media on sleep and guide targeted interventions. Encouraging responsible or healthy social media use is vital in reducing the risks of problematic use while highlighting the benefits as well.

## 1. Introduction

Social media usage is increasing rapidly and is becoming an integral part of daily life. Across global contexts, there is increasing availability and usage of digital devices, leading to heightened use of digital technology and social media by youth [1]. The published results of a study focusing on adolescents in 29 countries showed consistent problematic social media use across countries [2].

Social media is an important part of the emerging society. However, excessive social media use has been reported to increase depression, anxiety, mood, and loneliness [3]. The use of technology may also contribute to the perpetuation of sleep difficulties over the long term, through pathways such as heightened arousal, light exposure, time displacement, and nighttime interruption by technology devices [4]. As the world transitions to this new wave of technology dependence, it is vital to recommend safe and balanced social media use instead of discouraging or removing it completely. Additionally, the type of media content that an individual constantly engages with is what will continue to appear on their screen [5]. This cycle can influence self-image, emotions, and habits, including those connected to overall health and sleep [5]. As social media grows, it is important to examine the role it plays in overall health and well-being, especially in areas that affect human functionality like sleep. Sleep quality encompasses many aspects of sleep, including how satisfied you feel in your sleep, how easily you fall asleep, how well you stay asleep, how long you sleep, and how rested you feel when you wake up [6]. With the exponential usage of social media, reports are highlighting that young adults and adolescents are experiencing delayed sleep, daytime sleepiness, reduced sleep duration, poor sleep quality, and overall sleep deficiency [7]. There are many factors that may influence the relationship between social media usage and sleep quality. Some include blue light exposure from screens, which may disrupt sleep–wake cycles; constant notifications; habitual scrolling on platforms late at night; fear of missing out (FOMO); and insufficient rest [8,9,10], although one study has refuted the direct effect of short-wavelength light (perceived as blue-to-green light), suggesting the effects on sleep latency from a bright screen were minimal [4]. All these factors play a pivotal role in overall sleep quality.

Sleep deprivation has been associated with numerous health issues, both mental and physical. Two prime examples include lack of alertness and compromised immune regulation [11,12]. Additionally, sleep deprivation, whether partial or total, has been associated with impaired decision-making ability and increased risky decisions [13]. Therefore, sleep disturbances have significant consequences and warrant prompt attention. The relationship between social media and sleep quality is a growing concern across all age groups. However, for young adults and adolescents in particular, adequate sleep is crucial for their brain development and lifelong mental well-being [14]. Social media use has also been linked to mental health issues, poor sleep, academic difficulties, loneliness, and reduced self-esteem and life satisfaction [15]. Thus, among this cohort, the risks of poor sleep would have huge implications for their development, which warrants an investigation into the relationship between social media usage and sleep, particularly among this cohort. Therefore, this scoping review aims to further explore relationships between social media and sleep quality, particularly among adolescents and young adults.

Although many studies have examined social media use and sleep separately, there is still uncertainty about how strongly the two are connected. Some findings suggest that social media directly leads to poorer sleep, while others argue that individuals with poor sleep habits may simply spend more time online. These mixed outcomes highlight the need to review current evidence, identify consistencies and gaps in the literature, and point to directions for future research. By reviewing current systematic reviews and meta-analyses, this review also aims to summarize research, identify research gaps in the literature, and highlight areas of future research to address these critical issues.

## 2. Methodology

This scoping review of reviews was conducted using Arksey’s and O’Malley’s five-step framework [16] and the Preferred Reporting Items for Systematic Reviews and Meta-Analyses (PRISMA) extension for scoping reviews [17]. The completed PRISMA-ScR checklist can be found in the Supplementary Material. Arksey’s and O’Malley’s five stages include (1) identifying the research question; (2) gathering relevant studies; (3) selecting the studies; (4) charting the data; and (5) organizing, summarizing, and reporting the results [16]. There is no published protocol for this scoping review.

### 2.1. Identifying the Research Question

This scoping review of reviews explores the impact of social media use on sleep quality in adolescents and young adults. Thus, our research question was “What is the impact of social media use on sleep quality among adolescents and young adults?”

### 2.2. Gathering Relevant Studies

During the process of gathering relevant studies, a list of databases was used, which included PubMed, Web of Science, Embase, Medline, and Scopus. Using several databases allowed for a broader and more varied collection of research articles. Search terms were created to identify specific studies that aligned with our research question. The exact search terms used were (Social media) OR (Social networks) OR (Social platforms) OR (Digital media) OR (Social media platforms) OR (Social media networks) OR (Social media sites) OR (Social media apps) OR (Social networking apps) AND (Sleep) OR (time in bed) OR (nighttime rest) OR (bedtime) OR (insomnia) OR (fatigue) OR (circadian rhythm) AND (Adolescent) OR (young adult) OR (Youth) OR (Teenager) OR (Youngster) OR (Minor) OR (Young person) AND (Systematic review) OR (meta-analysis). Logical operators “OR/AND” were used to combine the search terms to help limit the data to match the study’s scope. The search was performed on 14 July 2025.

### 2.3. Selecting the Articles

Inclusion and exclusion criteria were used as a foundation in selecting the studies.

Studies were included if they (1) were a systematic review or meta-analysis, (2) were centered around adolescents and young adults, (3) discussed social media use in relation to sleep quality, (4) were peer-reviewed articles, and (5) were articles written in English. There was no restriction on publication year.

Studies were excluded if they (1) were primary articles, dissertations, or other non-review article types; (2) sampled adults; (3) were focused on non-social interactive media; (4) focused on screen time without specifying social media use; and (5) had a sample of individuals with pre-existing sleep disorders, such as insomnia.

### 2.4. Data Charting and Extraction

Articles were found using the selected databases and compiled to be imported into Covidence. Covidence is a web-based software platform designed to support systematic reviews. Covidence automatically removed duplicates, and two researchers independently reviewed the abstract and title and full texts to examine if they aligned with the given eligibility criteria. In addition, any conflicts regarding any articles based on the inclusion criteria were discussed by the two researchers until a consensus was reached.

Data were extracted from the included articles according to the following domains: author/date, study period, region of included studies, number and type of reviews, review aim, sample size and characteristics, risk of bias assessment, social media platforms examined, key findings on sleep quality, and future research recommendations.

### 2.5. Organizing, Summarizing, and Reporting the Results

All the data from the included articles are summarized in Table 1. Table 1 gives the descriptions and results reported in each article corresponding to the subject area under study. It also summarizes the identified research gaps in the articles exploring the relationship between social media and sleep quality.

## 3. Results

### 3.1. Article Search Selection

**Figure 1:**
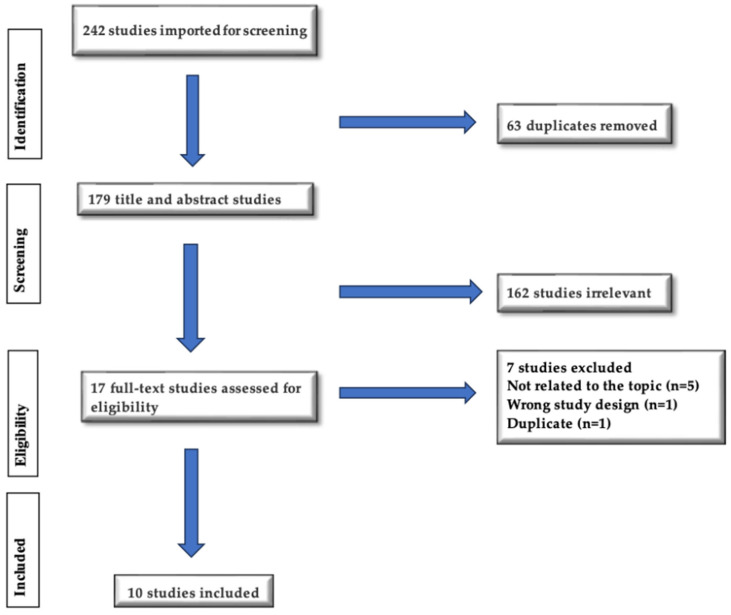
PRISMA flow chart.

After removal of duplicates and irrelevant studies, the search strategy yielded 17 out of 242 studies, which were screened at the full-text stage for inclusion. Two researchers collectively decided whether to include or exclude texts based on the previously established inclusion criteria. After screening, five studies were excluded since they were not related to the topic, one had the wrong study design, and one was a duplicate. Ten studies were included in the review. The PRISMA flow diagram (Figure 1) outlines the details.

### 3.2. General Review Articles Characteristics

In this scoping review of reviews, there were 10 articles that were selected in the final stages. Out of those selected, three were classified as meta-analyses, five were systematic reviews, and two were both. These articles were published from 2021 to 2024. From the 10 articles, a total of at least 998,552 participants were involved in these reviews. This approximation is due to the fact that some of the reviews did not list the number of participants involved in the individual studies they reviewed, focusing only on other characteristics of the participants and not the sample size.

Generally, the primary focus of our scoping review was on children, adolescents, and young adults, aged 0 to 32 years, and we included all review articles that focused on these cohorts. However, one article by Han et al. 2024 [22] was included because this review had a large sample size with a huge representation of children, adolescents, and young adults. The review also focused extensively on problematic social media use and sleep quality in this cohort despite the inclusion of a small representation of an adult population in the included primary studies.

The time periods represented in all these studies range from 1990 to 2024, and the geographical locations based on the continents of the primary studies were Asia, Europe, North America, Oceania, Australia, Africa, and South America.

### 3.3. Key Findings

In Table 1, the reviews showed that both general and problematic social media use was linked to poorer sleep quality and more sleep problems, though the effects were small and slightly significant [18,22]. Two reviews reported a general link between social media use and shorter sleep duration, but the results were inconsistent [18,21]. Moreover, problematic social media use, often measured by addiction scales, is shown to have a stronger negative impact on sleep than general use [18,22]. General social media use is usually measured by time spent on these platforms, and these discrepancies are found mainly in Eastern cultures and among younger users [20,22,23]. In a particular review, limited longitudinal evidence highlighted that poor sleep and frequent sleep problems may partly explain why excessive social media use is linked to worse mental health [19].

### 3.4. Recommendations

Many reviews suggest that participant details like age, gender, sample size, and statistical methods should be consistent to draw accurate conclusions based on the sample characteristics [18]. Objective assessments and validated tools are also recommended to improve tracking of media use and sleep [7]. Furthermore, research should explore how social media’s impact on sleep quality affects mental health and examine the psychological factors behind this connection [18]. One review suggested exploring diverse populations further with regard to gender differences and cultural factors, with a focus on children and adolescents [23]. There have also been suggestions of more comparisons between sleep and passive and active social media use and how brightness plays a role in affecting the relationship [21]. Moreover, more long-term studies with multiple check-ins and repeated measurements are strongly advised to understand the cause-and-effect relationship [24]. Highlighted mentions of potential causes of this effect were light exposure, circadian rhythm, type of content, and length of device use, all of which require more research to understand their roles [7], in particular, active social media use and how brightness plays a role in affecting the relationship [21]. Identifying teenagers most at risk and assessing the influence of parent management are also recommended to create interventions and prevent future health issues [24].

## 4. Discussion

### 4.1. Overview

This scoping review of reviews explored the link between social media use and sleep quality in adolescents and young adults. The results varied, with the majority highlighting a small but negative effect of social media use on sleep quality. Specific social media platforms, such as Twitter and Facebook, were linked to the most sleep disruptions, while Snapchat and Instagram had moderate effects, and WeChat had little to no effect, highlighting the complexity of the issue. The reviews considered several variables, including age, gender, and geographic disparities, which showed no clear findings and inconsistent patterns. These inconsistencies suggest that the role of social media in shaping sleep quality is not fully understood, and further research with consistent methodologies to assess the specific conditions under which social media affects sleep is warranted.

### 4.2. Social Media vs. Problematic Social Media

An important distinction should be made between the two prominent social media forms: general and problematic social media. In many articles, these categories of social media are investigated separately. The prevalence of problematic social media usage is difficult to navigate as there is no clear definition, leading to inconsistent methods of measurement [27]. The term is often used to describe excess social media use, marked by addiction-like features and/or lack of self-control that is linked to poorer mental health [18]. Problematic social media and social media addiction are used interchangeably, both characterized by behavioral addictive components [18,27,28]. In contrast, general social media usage, non-problematic social media, is based on social media use as a normal engagement with online platforms for social interaction, which can involve content sharing, networking, and communication for personal, social, or professional purposes [18]. Thus, the effect of social media on adolescents’ mental health has been suggested to be individualistic, shaped by patterns of usage and context specific [13]. The difficulty in quantifying social media makes it difficult to assess its impact on sleep.

A few but not all articles differentiate between these two types of social media usage, while some articles treated these two social media types as the same; this inconsistency affects how results can be analyzed across articles. Findings that combined general and problematic social media use showed weaker and irregular results as opposed to reviews that focused solely on problematic social media use, with stronger and more negative impacts on sleep. These comparisons are important because they highlight how very few studies distinctively compare the two types of use. Future studies should clarify whether interventions should focus on problematic social media or whether guidelines should adhere to the general use of social media.

### 4.3. Mental Health Regarding Sleep Quality and Social Media

Many of the articles reviewed showed a link between social media usage and sleep quality in relation to mental health. One article supported evidence that links excessive social media use to poor sleep quality and negative mental health in youth [19]. Mental health conditions, including depression, anxiety, and psychological distress, were explored [19]. The review concluded that longitudinal studies may partly explain why excessive social media use is linked to worse mental health, while cross-sectional studies can identify associations between variables but are limited in their ability to determine causality. Another article examined how sleep partly mediates the impact between social media use and externalizing behavior, and this was a bidirectional relationship [21]. In addition, Dibben et al. (2023) [21] reported that inadequate sleep plays a role in social media use and, later, mental health in both boys and girls but has a stronger impact on girls’ overall wellbeing. This suggests that sleep plays a key role in how social media affects both behavior and overall well-being. A key finding in a UK Millennium cohort study noted that general social media use was related to poor sleep, lower self-esteem, and negative body image, which were directly linked to increased depressive symptoms [29]. Regarding mental health, problematic social media was associated with depression and anxiety, while general social media use had a weak association [18]. However, it can be hard to understand if extensive social media use causes mental health issues or if people use social media as a maladaptive coping mechanism for existing mental health issues [19].

### 4.4. Social Media Use During COVID-19

The COVID-19 pandemic served as a period of isolation for many individuals. This event alone offered a unique opportunity to study the link between social media and sleep quality, as this period had an increase in digital technology use driven by social distancing measures and country-wide lockdowns [30]. The study reported mixed results: compulsive social media use was linked to sleep problems during and after lockdown, informed use was associated with sleep problems only during lockdown, and general use was linked to sleep disturbances after lockdown but not during it. This variety of results suggests that social media use affected sleep differently depending on the lockdown stage. Moreover, countries with stricter restrictions showed a stronger connection between social media use and sleep disturbances, highlighting that more confined environments may worsen the effects [26].

### 4.5. Positive Effects

No review showed a positive relationship between social media and sleep quality. All reported either no significant association or a negative association between the two variables. This is highly relevant because this may suggest that, while social media may not always be negative, it does not provide any benefits to sleep. Hence, the risks of using social media outweigh the positives, and limitations or safe-use practices should be encouraged.

### 4.6. Social Media and Dependency

Youth, ranging from children to young adults, are still in crucial stages of development [31]. Therefore, as social media is growing and is geared towards their engagement, it leads to a significant question of how vulnerable they are to these online platforms. As adolescents transition into adulthood, they are greatly affected by a social world where peer influence is magnified by social media and industries that exploit mental health [32]. Although social media per se is not bad and has positive attributes, excessive consumption of content can have negative effects. One potential reason why youth may be vulnerable to the effects of social media is due to social identity formation [28]. Social media allows for self-presentation, which is an important aspect of identity development, with a high chance of receiving immediate feedback from others [32,33]. Whether this feedback is positive or negative, it can influence what society considers attractive and how individuals perceive themselves. It can explain the need for adolescents and young adults to rely on these platforms to feel confident or up-to-date on what is the best evoked identity, possibly relating to their dependency and early problematic use.

### 4.7. Limitations and Future Recommendations

Limitations in this review include inconsistencies in the articles explored. The reviews examined different sample sizes, genders, age ranges, and geographical locations, making it difficult to draw consistent conclusions. Due to this lack of information, only broad and general conclusions could be made. Future studies require consistent methodologies and standardized definitions for objective assessment of sleep, diverse samples, and an improvement in the definition of the type of social media being explored. Additionally, only articles published in the English language were included in the review, leading to the potential exclusion of some eligible articles. All articles were retrieved from health and science databases, which may have introduced selection bias and led to the exclusion of relevant reviews indexed in other databases. Nevertheless, by focusing on systematic reviews and meta-analyses, this review captures a broader and more comprehensive overview of the existing literature.

### 4.8. Practical Implications and Policies

Although the results from this scoping review highlighted a small but significant effect of social media use on sleep quality, limiting further negative effects should always be encouraged. Practical measures such as interventions to monitor this relationship may be useful. One review reported that interventions, such as a targeted strategy of mobile phone restriction, when imposed an hour before bedtime among adolescents, led to “lights out” occurring 17 min earlier and an average increase of 19 min of total sleep per night [34]. This strategy may be implemented among adolescents and young adults to increase sleep duration. Educational institutions and school policymakers should promote awareness and provide targeted education on the impact of social media use on sleep. Additionally, educational programs should aim to raise awareness of potential risks associated with internet use, including the increased risk of cyber-victimization, and ensure healthy sleep is encouraged [21]. Furthermore, interventions categorizing different social media use and addressing ages susceptible to these effects can help address excessive use and improve sleep quality [23,26]. Therapeutic assessments can also be promoted to help those vulnerable to problematic social media use (PSMU) and included in educational programs to raise awareness about how social media use (SMU) influences wellbeing and mental health to encourage its use safely [18]. A systematic review on the impact of social media use interventions on mental well-being reported that therapy-based approaches, such as cognitive behavioral therapy, worked better, limiting or completely removing social media, with most of the therapeutic studies showing clear improvements [35].

## 5. Conclusions

The impact of social media use on sleep quality among adolescents and young adults showed a variety of results. The most consistent finding was that problematic social media use showed stronger effects than general use. The results were varied based on the social media platforms used, with Twitter and Facebook linked to the most sleep disruptions, while Snapchat and Instagram had moderate effects, and WeChat had little to no effect. While differences in primary study methods make it hard to draw firm conclusions, the results suggest that excessive social media use may contribute to shorter sleep duration, later bedtimes, and poorer sleep quality.

Future research should focus on longitudinal studies to better understand the lasting effects of social media use on sleep quality and guide targeted interventions. Future research should prioritize longitudinal designs in a controlled environment to have all participants partake in standardized assessment of sleep and social media use. Greater attention to cultural, gender, and age-related factors is also important to have a clear understanding of how these factors affect the results. Lastly, encouraging responsible social media use and raising awareness of healthy social media use are important for reducing the risks of problematic use while highlighting the benefits as well.

## Figures and Tables

**Table 1:** Data extracted from each article exploring social media impact on sleep.

Author/Date	Time Period Investigated	Continent/Countries of Primary Articles	Total Number of Included Articles/Review Types	Aim of Review	Sample Size/Characteristics	Risk of Bias Assessment	Type of Social Media Used	Key Findings on Impact on Sleep Quality	Recommendation for Future Research
Ahmed et al. 2024 [18]	Up to 2024	Asia, Europe, North America, Oceania	58 studies	To systematically assess the existing research and conduct a meta-analysis of the association between social media use and its potential negative effects on mental health and sleep concerning adolescents and young adults as well as to identify gaps and limitations from previous studies.	Social media use and sleep quality: 545,375 participants, weighted mean average 15 years old, female participants 50.7%. Problematic social media and sleep quality: 18,072 participants, weighted mean average 17 years old, female participants around 56%.	They took several steps to control and check any publication bias. Funnel plots were examined, and a statistical test, Egger Regression Test, was completed. They ran sensitivity analyses and used a covariance ratio to spot “influential studies” that might be skewing the results. They used the trim-and-fill method to estimate how missing extreme studies might change the results of the meta-analysis.	Facebook, Instagram, Twitter, and other social media platforms (WeChat, Snapchat, WhatsApp, YouTube, Google+, Vine, Tumblr, Pinterest, LinkedIn, and BlackBerry Messenger)	- Most cross-sectional studies link social media use to sleep problems, but longitudinal evidence is limited; overall, the effect is small and only slightly significant. - Many studies find a link between social media use and sleep duration, but combined results show a small, non-significant effect. - Problematic social media use consistently relates to sleep problems with a small meaningful effect, while its link to sleep duration is weaker and not significant; measurement methods affect findings more than demographics.	The recommendations for future research are that studies should consistently report participant details (age, gender, sample size, and statistical measures), investigate how different social media platforms uniquely affect sleep quality and mental health, and also conduct more longitudinal research with repeated measurements to clarify the direction of relationships between social media use and sleep. Exploring psychological factors such as motives for social media is recommended to examine how sleep mediates the relationship between problematic social media use and mental health outcomes.
Alonzo, Rea. 2021 [19]	Past 1990, up to 2024	27 studies from Asia, 9 from Europe, 3 from North America, 1 from Australia, and 1 spanned multiple continents	42	This review examines existing research on how active social media use relates to sleep quality and associates with common mental health issues among youth.	Age group of interest was age group 16–25 years; they included studies that used an age range of 12–30 years.	Most of the 42 studies showed low-to-moderate risk of bias. For the 6 cohort studies, most chose participants fairly, but some had issues with measuring social media use and sleep outcomes accurately. Three studies already had sleep or mental health problems present at the start, which could affect the results. Also, follow-up was not always complete, which might lead to bias. Most of the 36 cross-sectional studies recruited participants from schools or universities, so the results might not apply to all youth. Some studies had good participation and used trustworthy surveys, but many did not explain missing data well. Around half used poor methods to measure social media use, which could affect accuracy.	Interactive, active social media (e.g., Facebook)	- Longitudinal studies suggest that poor sleep and frequent sleep problems may partly explain why excessive social media use is linked to worse mental health. - Cross-sectional studies show mixed and complex relationships between social media use, sleep, and mental health, with effects going both ways.	Include studies that explore social use among different genders, assess the relationship between technologically populated groups, use consistent and objective measures to obtain more accurate results, and use long-term studies with diverse youth groups to further develop how social media use and sleep quality are connected.
Brautsch et al. 2023 [7]	Examined studies published from 1 January 2010 to 31 April 2021	Western countries include all EU member states along with Andorra, Iceland, Liechtenstein, Monaco, Norway, San Marino, Switzerland, Vatican City, Canada, the United States, Australia, and New Zealand	42 studies	The aim is to examine how digital media affects sleep given the rapid growth of digital technology. While previous research has documented the effects of digital media on children’s sleep, this study is investigating if similar effects occur in young adults (16–25 years).	The population studied consisted of individuals aged 16 to 25 years.	Many limitations were involved due to bias. It was reported that many of the studies sampled college students and self-referred participants, without showing clear age ranges. The studies in the review were quite different in their design, so it was not possible to combine their results in a meta-analysis. Most of the studies used self-reported data for digital media use and sleep, which may not be reliable due to memory errors or people wanting to give socially acceptable answers. Since many studies were cross-sectional, it is hard to say whether digital media use causes poor sleep. Also, a significant amount of the research did not look at what time of day media was used, which makes it difficult to understand how timing affects sleep. Few studies used objective tools to measure sleep or looked at gender differences, which are important areas for future research.	Digital media; (general communicative social media)	The studies found that social media is linked to shorter sleep duration and poorer sleep quality.	Use stronger experimental or longitudinal studies to clarify if digital media causes sleep changes, including effects of light and content on circadian rhythms. Apply objective sleep measures and validated tools for tracking media use. Study a wider range of media activities and how content activates or relaxes users. Examine how timing of media use affects sleep across the day and night. Investigate gender differences and other factors that may influence this relationship.
Chen et al. 2024 [20]	Up to 18 February 2024	Not stated	40 studies	To examine the overall relationship between problematic social media use (PSMU) and sleep quality and to assess how factors like age, gender, culture, and social media platform type may influence this relationship.	Total of 34,441 participants (no specific age range stated). Inconsistent characteristics of population (age, gender, and individualism/collectivism).	Many tests were taken to limit publication bias: funnel plots, Egger’s Regression Test, trim-fill method, and the class fail – safe N. Based on these tests, it concluded that the publication bias did not greatly influence the study’s results.	Online platforms (including Facebook, Instagram, Twitter, etc.)	- Younger users experienced worse sleep quality linked to problematic social media use. Facebook had the strongest impact on sleep, followed by Twitter, Snapchat, and Instagram. - WhatsApp and WeChat had smaller effects.	Future recommendations included investigating clinical populations, studying social features in games and streaming media, validating measurement scales for PSMU, using objective sleep measures, and accounting for temporal and geographical factors.
O. Dibben et al. 2023 [21]	Reviewed in October 2022	7 studies in Europe, 8 in North America, 8 in Oceania, and 5 in Asia	28 studies included	To combine longitudinal and experimental findings on how device use affects adolescents’ sleep and their mental health.	- The median sample size was 837.5. - Age range of study is 10–19 years; mean age being 14.8 years. - Median percentage of female participants across all studies is 52% (46–83%).	Risk of bias assessment was assessed in several categories, including high risk bias, selection bias, performative bias, detection bias, and attrition bias.	Software accessible through IED (ex: social media), passive or active	Many of the results did not find a clear correlation between social media use and poor sleep —there were inconsistent findings.	Further research should examine IED and social media use on sleep compared to passive screen activities, explore how diverse population factors modify sleep effects, and investigate the impact of screen brightness and length of device use on sleep outcomes.
Han et al. 2024 [22]	January 2018 to October 2023	Europe, Asia, Arabia, Oceania, North America, Africa, South America	55	To compare and analyze different types of digital media (phones, games, and social media) on sleep quality.	41,716 participants. Age range of general media used and sleep quality: 9.9 to 44 years (21,594).	Funnel plots were used to check for result imbalances, and a p-curve test was conducted to detect potential p-hacking. Both methods help measure and restrict publication bias.	General social media; specifically stated were Facebook and Twitter	- Social media use (general and problematic) is linked to poorer sleep quality and more sleep problems. - Findings show that problematic social media use (measured by addiction scales) affects sleep more negatively than general use (measured by time spent), especially in Eastern cultures.	More long-term and experimental research is needed to understand how electronic media use affects sleep quality, including cultural differences that might affect this relationship.
Lund et al. 2021 [23]	1 January 2009 to 31 August 2019	Western countries	49	To systematically review existing research on how electronic media use affects sleep in children and adolescents.	Children aged 0–15 years without any diagnoses or diseases.	The Effective Public Health Practice Project was used to assess risk of bias across 5 domains: selection bias, study design, confounders, data collection methods, and withdrawals and dropouts.	General social media	An association with social media and poor sleep quality was found, with the most between ages 13 and 15 years.	Investigate how sociodemographic factors impact media use on sleep in children and adolescents.
Pagano et al. 2023 [24]	Up to 28 January 2023	43.3% of the studies in Europe, 30.4% in North America, 4.4% in Asia (Korea, Kuwait, Iran, Taiwan), and 8.7% in Asia (China)	23	The aim is to explore how various digital media use is linked to sleep health.	116,431 participants; 52% were female, and the average age was 13.4 years.	To evaluate publication bias, funnel Plot and Egger’s Regression Test were conducted.	General social media	Results showed that social media has a small but negative effect on teen sleep. Apps like Twitter and Facebook were linked to shorter sleep duration, later bedtimes, and poorer sleep quality but not nearly to the same extent as traditional media.	Recommendations include long-term studies with multiple check-ins to understand how social media affects sleep and what factors influence this relationship. Future research should identify which teenagers are most at risk for poor sleep, especially considering things like their sleep patterns and how their parents manage their social media use.
Kaur et al. 2021 [25]	Not stated	21 countries across 7 geographical regions, data missing from Australasia and South America (specific countries/continents not stated)	43 studies	To investigate the link between social media use and sleep disturbances during the pandemic as well as factors that may influence this relationship.	68,247 residents. Across the included studies, participants had a mean age of 26.4 years, with males comprising 36% of the sample. In total, the studies represented 21 countries spanning seven geographical regions.	Publication bias was assessed using meta-regression, the R-index for replicability, and Egger’s test for small-study bias, and p-curve analysis for evidential value and potential p-hacking.	Types of social media mentioned are general, information focused, and compulsive social media	Compulsive social media use had a stronger association with sleep disturbances during and after the lockdown. Information-focused use had a positive association with sleep disturbances only during lockdown and not after. General use was linked to sleep disturbances after but not during lockdown.	Future recommendations include considering age-specific effects, comparing effects during versus after events like pandemics to capture changing patterns of use and sleep outcomes, and studying challenges during COVID-19 to improve future health emergencies.
Cheng et al. 2024 [26]	2013–2019	Australia, USA, UK, Bangladesh, China, Indonesia, The Netherlands, Estonia, Finland, Iran, Peru, Saudi Arabia, Scotland, Singapore	45	This review aims to examine how social media use at night effects sleep and related problems.	Examined adolescents (11–15 years) and young adults (16–32). Reviewed studies that included predominantly female participants.	The risks included are self-reported data, only cross-sectional data, convenience sampling, generalizability limits, and a lack of moderating variables.	Social media platforms (not specifically stated which type)	Social media users reported poor sleep, problematic sleep, and negative effects on individual health and performance efficiency.	Future research should explore how social media affects sleep through factors that influence the relationship, diversify research design, and include a wider range of participants.

## Data Availability

No new data were created or analyzed in this study.

## Supplementary Materials

The following supporting information can be downloaded at: https://www.mdpi.com/article/10.3390/children13010051/s1, Preferred Reporting Items for Systematic reviews and Meta-Analyses extension for Scoping Reviews (PRISMA-ScR) Checklist.
